# Assessing Local Health Department Performance in Diabetes Prevention and Control — North Carolina, 2005

**Published:** 2009-06-15

**Authors:** Deborah S. Porterfield, Janet Reaves, Marcus Plescia, Thomas R. Konrad, Bryan J. Weiner, Mary Davis, Edward L. Baker, Joanne M. Garrett, Curtis W. Dickson, Janet Alexander

**Affiliations:** RTI International; North Carolina Division of Public Health, Raleigh, North Carolina; North Carolina Division of Public Health, Raleigh, North Carolina; Cecil G. Sheps Center for Health Services Research, University of North Carolina, Chapel Hill, North Carolina; School of Public Health, University of North Carolina, Chapel Hill, North Carolina; School of Public Health, University of North Carolina, Chapel Hill, North Carolina; School of Public Health, University of North Carolina, Chapel Hill, North Carolina; School of Medicine, University of North Carolina at Chapel Hill, Chapel Hill, North Carolina; Hertford County Public Health Authority, Winton, North Carolina; Hertford County Public Health Authority, Winton, North Carolina

## Abstract

**Introduction:**

To improve the public health system's ability to prevent and control chronic diseases, we must first understand current practice and develop appropriate strategies for measuring performance. The objectives of this study were to measure capacity and performance of local health departments in diabetes prevention and control and to investigate characteristics associated with performance.

**Methods:**

In 2005, we conducted a cross-sectional mailed survey of all 85 North Carolina local health departments to assess capacity and performance in diabetes prevention and control based on the 10 Essential Public Health Services and adapted from the Local Public Health System Performance Assessment Instrument. We linked survey responses to county-level data, including data from a national survey of local health departments.

**Results:**

Local health departments reported a median of 0.05 full-time equivalent employees in diabetes prevention and 0.1 in control. Performance varied across the 10 Essential Services; activities most commonly reported included providing information to the public and to policy makers (76%), providing diabetes education (58%), and screening (74%). The mean score on a 10-point performance index was 3.5. Characteristics associated with performance were population size, health department size and accreditation status, and diabetes-specific external funding. Performance was not better in localities where the prevalence of diabetes was high or availability of primary care was low.

**Conclusion:**

Most North Carolina local health departments had limited capacity to conduct diabetes prevention or control programs in their communities. Diabetes is a major cause of illness and death, yet it is neglected in public health practice. These findings suggest opportunities to enhance local public health practice, particularly through targeted funding and technical assistance.

## Introduction

As noted in reports by the Institute of Medicine and others (1-3), as well as in a growing body of research (4,5), the US public health system is not adequately addressing current population health challenges. The September 11 attacks, anthrax dispersal, and Hurricane Katrina have focused attention on preparedness and bioterrorism issues, and substantial federal resources have flowed to state and local agencies to address gaps in personnel and programs. Although there is some evidence that these new dollars have increased capacity and performance across the board in the functioning of public health systems (6,7), others are concerned that existing resources at federal, state, and local levels have also been shifted to accommodate the current, urgent priority of preparedness (8).

Less visible public health challenges are the epidemics in chronic diseases, such as obesity and diabetes (9). Chronic diseases cause 70% of deaths in the United States and affect 90 million people (10). Yet chronic disease prevention and control in public health practice have been neglected, probably because of the historical roots of public health in addressing acute, infectious illnesses, the mechanisms of public health funding, and the possible perception that chronic diseases are not amenable to public health action (11). Limited evidence suggests that chronic disease programs and services in local public health lag behind the historically important issues of control of infectious diseases, including sexually transmitted diseases and tuberculosis; maternal and child health; and environmental health (12).

To improve the public health system's ability to prevent and control chronic diseases, it is necessary first to understand current practice and then to develop appropriate and valid strategies for measuring performance. Among the chronic diseases, diabetes is an optimal choice for studying the performance of governmental public health agencies in chronic disease prevention and control. The nation is facing an epidemic in type 2 diabetes and its related risk factor, obesity (9), and diabetes is widely recognized as a public and population health issue (13). Diabetes has also been a model for studying quality of care in the clinical setting, and well-accepted performance measures exist for the clinical setting (14) as well as evidence-based recommendations for both clinical and population services (15,16). Finally, public health funding and evaluation for diabetes programs through the Centers for Disease Control and Prevention (CDC) has a long history (17).

The objectives of our study were to measure capacity and performance in diabetes prevention and control in local health departments (LHDs) and to understand the characteristics of the LHD and the community that are associated with performance. The study was a collaboration among investigators at the University of North Carolina at Chapel Hill (UNC-Chapel Hill), the North Carolina Division of Public Health, Diabetes Prevention and Control Program (NC DPCP), and the North Carolina Association of Local Health Directors. North Carolina has a decentralized local public health system: the LHDs are overseen by local government and local boards of health and are independent of the state health department. A state health department grant program, Diabetes Today, provides funding to some LHDs, but otherwise LHDs receive no specific funding for public health activities related to diabetes.

## Methods

### Sample and survey method

In 2005, a cross-sectional mailed survey of all 85 LHDs (representing all 100 counties) in North Carolina was conducted to assess capacity and performance in diabetes prevention and control. The targeted respondent was the health director or his or her designated staff person working in diabetes. The mailed survey was preceded by an e-mail version of the survey cover letter and was followed by a reminder postcard and telephone call, a second mailing of the survey and second reminder postcard, and follow-up phone calls. Collaborators in the NC DPCP and the NC Association of Local Health Directors signed the initial cover letter and made several contacts with LHD directors to increase the response rate. As an incentive, each responding LHD was entered into a lottery for a scholarship for 1 person to attend a 5-day training in diabetes offered by a North Carolina university, worth approximately $850. The institutional review boards of the NC Division of Public Health and UNC-Chapel Hill approved the protocol.

### Measurement of key variables

The key variables of interest in the study were capacity and performance. We defined capacity as the number of full-time equivalent personnel (FTEs) in diabetes prevention or control, and performance was defined as the self-reported provision of a diabetes-specific service or program. Questions were based on the 10 Essential Public Health Services (monitor, diagnose and investigate, inform and educate, mobilize, develop policies and plans, enforce, link, assure, evaluate, and research) and adapted from the Local Public Health System Performance Assessment Instrument developed by the National Public Health Performance Standards Program at CDC (18). This instrument, first released in 2002, provides a mechanism to measure generic (rather than disease-specific) performance of a local public health system. We adapted items from the CDC instrument to make them diabetes-specific, and we included new questions developed to measure performance of specific diabetes-related programs or services. Steps taken to ensure that the survey was relevant to local public health practice and inclusive of all diabetes-related services being offered by LHDs were 1) review of the proposed questions by staff at the NC DPCP and at CDC, 2) review of the proposed questions by the Health Promotion Committee of the NC Association of Local Health Directors, and 3) pilot administration of the survey to representatives from 3 NC LHDs who were recruited by the investigators. The pilot led to only minor revisions and clarifications of the survey. We assessed validity of the survey during the pilot phase; pilot respondents were asked whether the proposed indicators adequately measured capacity and performance and whether the proposed indicators were complete.

We linked survey responses to secondary data to assess the characteristics of the health departments and the jurisdictions that were associated with high performance. The characteristics of interest were based on a model of public health system performance (19) and a review of the public health systems research literature. Specifically, we incorporated from secondary sources additional variables that have a previously demonstrated association with global LHD performance: staffing levels, expenditures, type of jurisdiction, and population characteristics such as size and poverty rate (4,20-23). Additional characteristics of the LHD that we examined were the presence of a full-time medical director and the presence of “diabetes” or “chronic disease” in the mission statement. We also considered whether the LHD had a history of diabetes-specific funding through 2 external sources known to the authors: 1) Diabetes Today grants from the NC DPCP for development of local diabetes programs, and 2) Project IDEAL (Improving Diabetes Education, Access to Care, and Living), a 1-time grant program of a local foundation to enhance delivery of health care services for underserved people with diabetes. Other characteristics considered were the jurisdiction (whether the jurisdiction was part of or contained a metropolitan statistical area), the prevalence of diabetes in the jurisdiction, the presence or absence of a community or migrant health center or free clinic, and the physician-to-population ratio. We also examined the relationship of performance to the department’s accreditation status, although accreditation status, at the time a voluntary process, was not considered part of a causal pathway. Additional data sources used were the NC DPCP (diabetes prevalence and history of Diabetes Today funding) (E. Valeriano, MD, unpublished data, February 2006; C. Haynes-Morgan, written communication, December 2005), the NC Community Health Center Association (presence of a community or migrant health center) (24), the US Census (population, poverty level, and relationship to metropolitan statistical areas) (25,26), the NC Health Professions Data System (physician-to-population ratio) (27), the NC Institute for Public Health (accreditation status) (28), and the 2005 survey of LHDs conducted by the National Association of County and City Health Officials (LHD FTEs and expenditures) (12).

### Data analysis

To report capacity and performance in diabetes prevention and control, we present simple univariate descriptions of item responses. The study had a secondary objective of exploring the characteristics of the health departments and jurisdictions that were associated with high diabetes-related performance. To do this efficiently, we created a summary performance score, which was a simple index of performance based on the 10 essential services. A total of 33 yes/no questions assessing key programs or services were used to assign a score (between 0 and 1) for each essential service. The score represented the average of 1 to 5 questions per essential service; in the event of a missing response to a question (≤3 missing values [4%] for all questions), the remaining responses were averaged for that essential service. Subsequently, the scores for each of the 10 essential services were summed to create an index of total performance, with a range of 0 to 10. Bivariate analyses (*t* tests and Spearman correlation coefficients), using a cutoff for significance of *P* < .05, and multiple linear regression were conducted to investigate which independent variables were associated with the outcome of the performance index. Because the sample size was small, the effect of confounding was assessed 1 variable at a time.

The independent variables of main interest were history of diabetes-specific external funding (Diabetes Today or Project IDEAL), presence of a stated diabetes or chronic disease-related mission statement, and estimates of need for diabetes-related programs (high prevalence, low capacity of medical care delivery system). The relationship of general structural capacity measures (such as size, general staffing, and total expenditures) to performance was investigated, but in the modeling they were considered potential confounders. We investigated as other confounders the demographic characteristics of the jurisdiction, such as poverty rate and urban or rural status.

## Results

The response rate was 100%. Forty-six LHDs received a second mailing of the survey, and 8 LHDs requested a third copy of the survey on follow-up telephone calls. Survey responses were obtained over the telephone at the request of the LHD in 3 instances. On average, 2.2 people were involved in completing each survey on behalf of the LHD. The most common respondents were nurses, followed by health educators, health directors, and nutritionists. Health directors directly participated in 25% of the responses.

The median number of FTEs per LHD was 80, and the median yearly expenditures were $4.81 million (Table 1). Slightly more than one-third (35%) of LHDs had received diabetes-specific funding through Diabetes Today, and only 3 (4%) had received funding through Project IDEAL. Only 19% had "chronic disease" or "diabetes" in their mission statement. Almost half the jurisdictions were urban, defined as containing a metropolitan statistical area within the jurisdiction, and nearly one-third had a population of more than 100,000.

Health departments reported limited capacity in diabetes: the median number of FTEs was 0.05 in prevention (interquartile range [IQR], 0-0.5), 0.1 in control (IQR 0-0.5), and 0.3 in prevention or control (IQR 0-1.0) (data not shown). Forty percent reported no FTEs devoted to diabetes prevention or control. In terms of specific provider types, only 16% reported having a certified diabetes educator on staff. The most common provider types reported by LHDs were nurses, followed by nutritionists, health educators, nurse practitioners or physician assistants, and physicians. Only 12% reported any physician FTEs devoted to diabetes prevention or control.

Self-reported performance varied widely across the essential services (Table 2). Most LHDs reported access to data on diabetes prevalence (87%) and risk factors (70%), and many reported monitoring changes in these prevalences. Other activities commonly reported by LHDs included providing information to the public and policy makers, using media to communicate diabetes health information, providing health education for people with diabetes, and screening for diabetes and prediabetes.

Other programs and services were reported less commonly. Only half had a coalition or committee that focuses on diabetes. Less than half reported assessing the extent to which primary care or diabetes education was available in their community, and only 11% reported conducting a recent diabetes-related public and personal workforce assessment. Other activities less commonly reported involved public policy; training for health care providers; modification of laws, regulations, or ordinances; research; and evaluation.

The mean score for all LHDs on the 10-point index of performance was 3.5 (SD = 1.9). Of the main characteristics of interest, only the history of diabetes-specific external funding (Diabetes Today or Project IDEAL) was associated with performance (Table 3). LHDs with a history of funding from Diabetes Today had a mean index of 4.1 compared with 3.2 for those without (*P* = .03). LHDs with a history of funding through Project IDEAL had a mean index of 6.7 compared with 3.4 for those without (*P* = .002). Measures of need (diabetes prevalence, presence of a community or migrant health center or free clinic, and physician-to-population ratio) were not associated with performance, nor were having "diabetes" or "chronic disease" in the mission statement. Population size of the jurisdiction and LHD size (measured by FTEs or expenditures) were also associated with performance. Health departments that had received accreditation were also more likely to have a high total performance score.

Multiple linear regression was used to investigate whether the observed association between Diabetes Today funding and the performance index was confounded by other factors. (All 3 LHDs with Project IDEAL funding had also received Diabetes Today funding). Complete data on FTEs and expenditures were not available (because of the item response rate of the Profile survey [12], the source for those variables), and therefore, these could not be used in the model as measures of health department size. Instead, we used population size of the county as a surrogate measure because this measure was highly correlated with FTEs (0.89). However, controlling for population size did not change the association between Diabetes Today funding and the performance index. We also assessed the following variables as potential confounders, and none had any effect on the relationship of Diabetes Today funding to the performance outcome: presence of any community or migrant health center or free clinic, physician-to-population ratio, rural or urban status, percentage of population below the poverty level, or diabetes prevalence.

## Discussion

This survey of North Carolina LHDs found that most have limited capacity to conduct or coordinate diabetes prevention or control programs in local communities. Self-report of some programs and services was high, particularly in areas such as surveillance, health education, and screening. However, we found limited performance in other areas such as assessing availability of health services or health education for people with diabetes or participating in public policy. One finding was that total performance of the LHD was not higher in areas of greater need (higher diabetes prevalence or lower capacity of the medical care delivery system). A history of diabetes-specific external funding was associated with LHD performance even when controlling for potential confounders such as LHD size.

This is the first study known to the authors to measure performance of LHDs in a chronic disease. Previous studies of LHDs have focused on measuring global performance (20,21,23), preparedness (29), or maternal and child health. Although global performance studies may be more germane for long-term performance measurement (30), this study provides a key insight into the lack of programs for an important chronic disease. More work is needed to measure LHD performance in other chronic disease areas, such as obesity, cardiovascular disease, and cancer prevention and control. In addition, a comprehensive, integrated assessment of prevention and control activities for all of the major chronic diseases would provide a fuller picture of how LHDs are able to address chronic disease than this study can provide.

This study and the survey itself do, however, provide an important example of how a state program (the NC DPCP) can measure LHD performance for evaluation and program improvement and measure the effect of its grants to LHDs. A similar instrument, developed by the Diabetes Council of the National Association of Chronic Disease Directors (31), exists for state programs to measure the performance of the state public health system. Although results must be interpreted cautiously, the data offer some evidence for the effectiveness of the funding and technical assistance that the NC DPCP provides to LHDs through the Diabetes Today program. A more comprehensive evaluation, including how the Diabetes Today model is implemented in different states, appears warranted. The data also point out areas where additional technical assistance is needed, for example, gaps noted between LHD programs and certain evidence-based practices such as screening or provision of diabetes education.

The findings that LHD size or population size and LHD funding affect performance are consistent with those of other studies (4,20,21). Unlike in studies of LHD performance in other areas of disease prevention and control, poverty rate (4) or type of jurisdiction (21) was not related to performance. This finding may be due to the sample size, the range of variation in these variables in the state examined, or features unique to the development of diabetes programs. One finding was that need of the jurisdiction was not associated with LHD performance, and this attempt to examine the association is rare in public health performance literature.

This study represents a snapshot of all possible types of diabetes-related programs and services, not necessarily those that are most important to local public health practice. The index itself is weighted to represent each essential service equally, which may also not be appropriate. Key informants and stakeholders should be interviewed to refine the instrument by identifying which items are the priorities for LHDs.

### Limitations

This study has several limitations. Because the data are self-reported, performance may be overreported. Almost all studies of LHD performance rely on self-reported data. In addition, variation in numbers and types of staff responding to the survey may have introduced some measurement error. Limitations of individual survey items included that the survey did not assess amount, reach, or quality of programs, only the presence or absence of programs. In addition, the amount or duration of diabetes-specific external funding was not available. The most important limitation, however, is that the performance index has not been formally validated. Replication of this work in other states and studies to validate the instrument are needed. With respect to the associations between LHD characteristics and performance, this is a cross-sectional study, and no determinations of causation can be made. The sample size was limited, and results from North Carolina may not be generalizable to other states, especially those that are outside the Southeast or that do not have a decentralized LHD structure.

The survey also did not measure characteristics of LHDs that are likely predictors of diabetes-related capacity and performance, for example, the extent or quality of partnerships of the LHD; the nature of leadership within the LHD; and organizational climate, especially as it pertains to adoption of evidence-based recommendations or guidelines. A follow-up study, which consists of case studies of high-performing LHDs, will allow investigation of these hypotheses. Finally, although not necessarily a limitation of the study, the outcome measured in this study, as in most studies of public health performance, was the performance of the LHD alone and not the local public health system. Local health department performance, here measured as the presence of certain programs or services, may vary on the basis of what is otherwise available in the service area.

### Conclusion

This study documents the low level of capacity and performance in diabetes prevention and control among NC LHDs. Despite the well-described threats of the diabetes and obesity epidemics to the nation's health, LHDs may not be well positioned to conduct or coordinate effective diabetes prevention or control in most communities. This study, although cross-sectional in design, also suggests that external funding is critical for building programs to address chronic disease and the need of a community may not necessarily determine the programs or services that are offered. Targeted funding offers the opportunity to develop a local public health system that can address the less visible but urgent chronic disease challenges of our time.

## Figures and Tables

**Table 1:** Characteristics of Local Health Departments and Their Jurisdictions (N = 85) — North Carolina, 2005

**Characteristic**	No. of Departments^a^	Value
**Health department**		
No. of full-time equivalent employees, median (interquartile range [IQR])	73	80 (51–128)
Expenditures, in millions of dollars, median (IQR)	74	4.81 (2.85–8.03)
Accredited^b^, frequency (%)	85	20 (23.5)
Has a full-time medical director, frequency (%)	85	17 (20.0)
Has received Diabetes Today^c^ training or funding, frequency (%)	85	30 (35.3)
Has received Project IDEAL (Improving Diabetes Education, Access to Care, and Living)^d^ funding, frequency (%)	85	3 (3.5)
Diabetes or chronic disease in mission statement, frequency (%)	74	14 (18.9)
**Jurisdiction**		
Single-county, frequency (%)	85	79 (92.9)
Metropolitan statistical area, frequency (%)	85	40 (47.1)
Population >100,000, frequency (%)	85	26 (30.6)
Percentage of population below poverty level, mean (SD)	85	14.0 (4.2)
Contains a community or migrant health center, frequency (%)	85	60 (70.6)
Physicians per 100,000 population, median (IQR)	85	62.0 (47.8–89.0)
Estimated diabetes prevalence, mean (SD)	85	9.1% (0.93)

a Number of local health departments with available data, either from the study survey or from *2005 National Profile of Local Health Departments* (12).

b Accredited through a voluntary process by the North Carolina Local Health Department Accreditation Board.

c Grants from the North Carolina Diabetes Prevention and Control Program for development of local diabetes programs.

d A 1-time grant program of a local foundation to enhance delivery of health care services for underserved people with diabetes.

**Table 2:** Programs and Services in Diabetes Prevention and Control in Local Health Departments (N = 85), by Essential Service^a^ — North Carolina, 2005

**Characteristic**	**No. of Departments** b	Frequency (%)
**Essential service 1 — monitor**		
Has conducted a community health assessment for diabetes	84	37 (44.1)
Has access to community data on		
Prevalence of diabetes	84	73 (86.9)
Prevalence of risk factors for diabetes	84	59 (70.2)
Availability of health resources for people with diabetes	84	65 (77.4)
Quality of diabetes care	83	28 (33.7)
Health status	83	23 (27.7)
**Essential service 2 — diagnose and investigate**		
Monitors changes in diabetes prevalence and risk factors	84	37 (44.1)
Has access to a master's- or doctoral-level epidemiologist	84	18 (21.4)
Has access to laboratories capable of meeting routine surveillance and diagnostic needs	84	61 (72.6)
**Essential service 3 — inform and educate**		
Provides public and policy leaders with information on diabetes and its risk factors	84	64 (76.2)
Uses media to communicate health information	84	53 (63.1)
Uses materials by National Diabetes Education Program	81	54 (66.7)
Sponsors health education programs for people with diabetes	83	48 (57.8)
Sponsors health education programs for people at risk for diabetes	71	44 (62.0)
Conducts health promotion programs for people with or at risk for diabetes	82	53 (64.6)
**Essential service 4 — mobilize**		
Uses communication strategies to strengthen links or inform constituents about diabetes	83	39 (47.0)
Has a coalition or committee that focuses on diabetes	84	44 (52.4)
**Essential service 5 — develop policies and plans**		
Has been involved in activities that influenced or informed the public health policy process in diabetes prevention and control	83	14 (16.9)
Issues briefs	85	6 (7.1)
Provides public testimony	85	4 (4.7)
Participates on advisory board	85	9 (10.6)
Meets with elected officials	85	6 (7.1)
Has established a process for community health improvement in diabetes	84	20 (23.8)
Has a community health improvement plan for diabetes	82	15 (18.3)
**Essential service 6 — enforce**		
Participated in the last 5 years in development or modification of laws, regulations, or ordinances	82	7 (8.5)
**Essential service 7 — link**		
Has assessed the extent to which personal health services are accessible, acceptable, and available	84	34 (40.5)
Has assessed the extent to which diabetes education is accessible, acceptable, and available	84	39 (46.4)
Provides primary care to people with diabetes	84	31 (36.9)
Maintains registry of diabetes patients	30	8 (26.7)
Provides case management	84	29 (34.5)
Provides disease management	84	26 (31.0)
Screens for diabetes	83	61 (73.5)
Screens for prediabetes	84	40 (47.6)
**Essential service 8 — assure**		
Conducted a public and personal workforce assessment in last 3 years	84	9 (10.7)
Anyone in LHD has attended a diabetes-related training or conference in last 3 years	85	42 (49.4)
Has conducted trainings for health care providers in community in the last year	84	8 (9.5)
**Essential service 9 — evaluate**		
Has evaluated population-based health services in last 3 years	84	9 (10.7)
Has evaluated personal-based health services in last 3 years	84	7 (8.3)
**Essential service 10 — research**		
Identifies or monitors best practices	82	42 (51.2)
Involved in research studies	82	4 (4.9)

a Measures were based on the 10 Essential Public Health Services and adapted from the Local Public Health System Performance Assessment Instrument developed by the National Public Health Performance Standards Program at the Centers for Disease Control and Prevention (18).

b Number of local health departments that responded to the question.

**Table 3:** Associations Between Health Department or Jurisdiction (N = 85) Characteristics and 10-Point Mean Performance Index^a^ — North Carolina, 2005

**Characteristic**	No. of Departments^b^	*R* c	Mean Index	*P* value^d^
**Health department**				
Median no. of full-time equivalent employees	73	0.349	—	.003
Median expenditures, in millions of dollars	74	0.363	—	.002
Accredited				
Yes	20	—	4.3	.025
No	65	—	3.2	
Has a full-time medical director				
Yes	17	—	3.9	.31
No	68	—	3.4	
Has received Diabetes Today^e^ training or funding				
Yes	30	—	4.1	.03
No	55	—	3.2	
Has received Project IDEAL (Improving Diabetes Education, Access to Care, and Living)^f^ funding				
Yes	3	—	6.7	.002
No	82	—	3.4	
Diabetes or chronic disease in mission statement				
Yes	14	—	3.4	.83
No	60	—	3.5	
**Jurisdiction**				
Single-county jurisdiction				
Yes	79	—	3.4	.34
No	6	—	4.1	
Metropolitan statistical area				
Yes	40	—	3.6	.72
No	45	—	3.4	
Population >100,000				
Yes	26	—	4.3	.01
No	59	—	3.1	
Percentage of population below poverty level	85	0.126	—	.25
Contains a community or migrant health center or free clinic				
Yes	60	—	3.6	.56
No	25	—	3.3	
Mean no. of physicians per 100,000 population (mean)	85	0.015	—	.89
Mean estimated diabetes prevalence (mean)	85	–0.120	—	.28

a For each local health department, responses to 33 questions, which were adapted from the Local Public Health System Performance Assessment Instrument developed by the National Public Health Performance Standards Program at the Centers for Disease Control and Prevention (18), were combined to provide a score for each of the 10 Essential Public Health Services. The scores were then summed into a 10-point index of total performance.

b Number of local health departments with available data in each category.

c Spearman correlation coefficient.

d *t* tests for categorical variables and Spearman correlation coefficients for continuous variables.

e Grants from the North Carolina Diabetes Prevention and Control Program for development of local diabetes programs.

f A 1-time grant program of a local foundation to enhance delivery of health care services for underserved people with diabetes.
